# Expression of *Fragaria vesca PIP* Aquaporins in Response to Drought Stress: *PIP* Down-Regulation Correlates with the Decline in Substrate Moisture Content

**DOI:** 10.1371/journal.pone.0074945

**Published:** 2013-09-23

**Authors:** Nada Šurbanovski, Daniel J. Sargent, Mark A. Else, David W. Simpson, Hanma Zhang, Olga M. Grant

**Affiliations:** 1 Research and Innovation Centre, Fondazione Edmund Mach, San Michele all’Adige, Trentino, Italy; 2 East Malling Research, East Malling, Kent, United Kingdom; 3 Faculty of Biological Sciences, University of Leeds, Leeds, United Kingdom; 4 University College Dublin Forestry, University College Dublin, Dublin, Ireland; National Council of Research (CNR), Italy

## Abstract

*PIP* aquaporin responses to drought stress can vary considerably depending on the isoform, tissue, species or level of stress; however, a general down-regulation of these genes is thought to help reduce water loss and prevent backflow of water to the drying soil. It has been suggested therefore, that it may be necessary for the plant to limit aquaporin production during drought stress, but it is unknown whether aquaporin down-regulation is gradual or triggered by a particular intensity of the stress. In this study, ten *Fragaria PIP* genes were identified from the woodland strawberry (*Fragaria vesca* L.) genome sequence and characterised at the sequence level. The water relations of *F. vesca* were investigated and the effect of different intensities of drought stress on the expression of four *PIP* genes, as well as how drought stress influences their diurnal transcription was determined. *PIP* down-regulation in the root corresponded to the level of drought stress. Moreover, transcript abundance of two genes highly expressed in the root (*FvPIP1;1* and *FvPIP2;1*) was strongly correlated to the decline in substrate moisture content. The amplitude of diurnal aquaporin expression in the leaves was down-regulated by drought without altering the pattern, but showing an intensity-dependent effect. The results show that transcription of *PIP* aquaporins can be fine-tuned with the environment in response to declining water availability.

## Introduction

Drought is an environmental stress that produces a plant water deficit sufficient to disturb internal physiological processes [1]. In drying soil, the water potential decreases with the decreasing soil moisture content, reducing the amount of water available for the plant to absorb [2]. Physiological parameters of plant water balance, such as water potential, hydraulic resistance, stomatal conductance and transpiration, change in response to drought stress as various mechanisms start to operate in order to minimise water loss, maximise water uptake and improve plant water status.

Aquaporins are transmembrane proteins, members of the major intrinsic protein (MIP) family that facilitate the passive movement of water through cells and play a crucial role in plant water relations [3] [4] [5] [6]. Aquaporins have been shown to be involved in numerous physiological processes, particularly in water uptake and radial water transport [6] [7] [8] [9] [10], and there is now substantial physiological and genetic evidence that most of the short-term changes in root hydraulics are mediated through the regulation of aquaporin expression and activity [11]. Aquaporins have also been implicated in leaf water relations including mediating water transport from the xylem to the stomatal chamber [12] [13] and responding to different environmental factors including water stress, cold stress and irradiance [14].

Plant aquaporins are remarkably diverse, with several subfamilies of MIPs identified in dicots [15] [16]. The plasma membrane intrinsic proteins (PIP) and the tonoplast intrinsic proteins (TIP) subfamilies correspond to aquaporins that are abundantly expressed in the plasma and vacuolar membranes, respectively and represent central pathways for transcellular and intracellular water transport [4]. A third group, Nodulin26-like intrinsic proteins (NIP) are close homologues of GmNod26, an abundant aquaporin in the peribacteroid membrane of symbiotic nitrogen-fixing nodules of soybean roots, which are also present in non-leguminous plants where they have been localised in plasma and intracellular membranes [4]. Two additional subfamilies are also known but have have thus far been poorly characterised; small basic intrinsic proteins (SIP) [15] and the most recently identified subfamily X intrinsic proteins (XIP) [16].

Aquaporins have a common secondary structure consisting of six transmembrane α-helices (TM1-6) connected with five loops (A–D) of which loops B and E are hydrophobic and contain a small α-helix each, both ending with the highly conserved asparagine-proline-alanine (NPA) signature motif [17] [18]. This motif, alongside the aromatic/arginine selectivity filter, determines the substrate specificity of aquaporins [17] [19]. Because of their abundance in plant tissues, the plasma membrane intrinsic protein (PIP) and the tonoplast intrinsic protein (TIP) subfamilies are thought to play a key role in transcellular and intracellular plant water transport [4]. Whilst the tonoplast membrane is generally more permeable than the plasma membrane, the conductivity of isolated protoplasts has a broader range of values than isolated vacuoles, indicating that the control of transcellular water flow probably resides in the plasma membrane [20]. The PIP subfamily is the largest subfamily of plant aquaporins and has been further divided into two subgroups, PIP1 and PIP2. PIP2 isoforms have a shorter amino-terminal extension and a longer carboxy-terminal end than PIP1 isoforms, and from a functional perspective display more efficient water channel activity [21] [22], although PIP2 activity can be enhanced by PIP1 proteins [23] [24].

*PIP* aquaporins are involved in numerous physiological processes and are highly responsive to environmental stimuli. Many *PIP* genes display diurnal expression patterns [25] [26] [27] [28] [29]. In a study on roots of *Lotus japonicus*, the expression of *PIP1* genes peaked 6–8 hours after the onset of light and reached a minimum at the onset of darkness [25]. In *Vitis vinifera* roots, *VvPIP1;1* expression rose 3 hours after the onset of light and remained at the same level until darkness [24], whilst in *Pisum sativum* lateral roots and taproots had different patterns of expression, both with two distinct peaks during the day [28]. In maize, the expression of two *PIP1* and two *PIP2* genes in the root rose sharply 2–4 hours after the beginning of the photoperiod and was maintained under darkness for one day, after which the diurnal rhythm ceased [27]. The fluctuation of maize leaf *PIP* transcript abundance under normal photoperiod was concordant with the profile reported for the root [29]. The diurnal rhythm of aquaporin expression has been linked to important water balance parameters, such as changes in the root hydraulic conductance [25] [28] and transpiration [30] [31]. It is not known, however, how the daily rhythm of aquaporin expression is affected by environmental stresses such as drought.

In general, *PIP* aquaporin response to drought has been shown to vary considerably depending on the isoform, tissue, species and variety, the presence of symbionts or level of stress. In leaves of grapevine, moderate drought stress led to a significant decrease in expression whilst prolonged or increased stress caused an up-regulation of the five *PIP* genes investigated [32]. Another study showed that *VvPIP1;1* in the root was up-regulated by drought stress in an anisohydric but not in an isohydric cultivar of grapevine [24]. In a study of *Phaseolus vulgaris*, *PIP* genes responded differently to drought stress depending on whether the plants had been inoculated with arbuscular mycorrhizal fungi [33]. Strong down-regulation of *PIP* transcription under drought stress was observed in roots and twigs of olive [34], as well as in tobacco roots [23], and peach fruit tissue [35]. Several comprehensive studies in *Arabidopsis thaliana* have shown that most *AtPIP* aquaporins undergo a transcriptional down-regulation under drought and salinity stresses, whilst fewer genes were found to be up-regulated or maintained at the same level [36] [37] [38] [39]. Alexandersson *et al*. [37] showed that under drought stress, ten out of the thirteen Arabidopsis *PIP* genes were down-regulated at both transcript and protein levels. Only one of the isoforms (*AtPIP2;6*) was maintained at the same expression level and two genes (*AtPIP1;4* and *AtPIP2;5*) were shown to be up-regulated. All the *PIP* genes that were down-regulated by drought were highly expressed in the root system. The transcriptional response was conserved between different Arabidopsis accessions and the down-regulated genes were found to be strongly co-expressed, unlike the genes that were up-regulated or maintained at the same level [39]. Under drought stress conditions, root hydraulic conductivity, which is regulated partially by *PIP* aquaporins, declines − most probably as a mechanism to avoid water flow from root to soil whilst the soil water potential is decreasing [40]. General down-regulation of aquaporins is thought to help reduce water loss and prevent backflow of water to the drying soil [37] [30]. It has been suggested therefore, that it may be necessary for the plant to limit aquaporin production at certain levels of drought stress [37] but the question remains whether aquaporin down-regulation is gradual or triggered by a particular intensity of drought.

In recent years, drought stress has become an increasingly important problem in regions where it was negligible in the past. Considering that agriculture is one of the largest users of water, predictions that fresh water resources are expected to come under severe pressure in the future [41] emphasize the need for a detailed understanding of drought stress response in agriculturally important crop species. The genus *Fragaria* L., (strawberry) belongs to the Rosaceae, a family comprising over 100 flowering plant genera. Many Rosaceous species are cultivated fruit crops of high nutritional value and economic importance, which have considerable water consumption needs. The woodland strawberry (*F. vesca* L.) is a model plant and a versatile experimental system [42] [43] whose genome has recently been sequenced [44]. In contrast to the in-depth genetic and genomic studies performed on *F. vesca*, very little is known about plant water relations of this species, and no studies have investigated *F. vesca* aquaporins thus far.

We identified the *PIP* gene sequences present in the *F. vesca* genome and performed phylogenetic analyses to classify them in relation to previously described *PIP* genes in other plant species. Prior to the *Fragaria* genome sequence becoming available, four partial cDNA sequences of aquaporins were obtained from drought stressed *Fragaria* plants using degenerate primers designed from *Arabidopsis PIP* sequences. As these genes were known to be expressed under drought stress, we investigated their expression in leaves in response to diurnal signals after four weeks of moderate drought stress and additionally, in roots and leaves after subjecting the plants to different levels of drought stress. One of the aims of the study was to establish whether diurnal expression of aquaporins changed under water stress conditions. Another goal was to determine the effects of different intensities of drought stress on *PIP* expression in *F. vesca*.

## Materials and Methods

### Plant Material, Experimental Conditions and Physiological Measurements

Six week old *F. vesca* plants, clonally propagated from stolons, were used in the study. Plants were potted in super-fine perlite (0–2 mm) with 3.0 kg m^−3^ of Osmocote Exact Mini 3–4 M controlled release fertiliser (Scotts Professional, the Netherlands) in 1 l pots. The pots were covered with non-transparent covers to prevent evaporation from the substrate. Plants were grown in the period July-August, in a controlled-environment compartment of a contained facility at 22–24°C during the day; 17–18°C during the night, with relative humidity at 60% during the day and 80% during the night. No artificial light was supplied; the average and maximum photosynthetically active radiation in the compartment was recorded (Figure S1) using a data logger (Data Hog 2 Skye instruments Ltd., Powys, UK). All plants were maintained under well watered conditions before treatments. Plants were irrigated with 100 g l^−1^ liquid feed solution (Agrosol 316 N:P:K 13∶5∶28) diluted to 0.15 g l^−1^ through a chemical injector (Dosatron DI 16, Dosatron International S.A., France) attached to the irrigation system.

Two experiments were conducted with the above set-up. In the first experiment, moderate water restriction was applied for four weeks, after which the plants were sampled diurnally for two days. In the second experiment, two levels of severe water deficit were applied for six days, after which the plants were watered for two days. Tissue was sampled on day six and day eight. In both experiments a randomised block design with each irrigation treatment replicated across four blocks was implemented. The control plants of both experiments were provided with sufficient irrigation to compensate for 100% of the evapotranspiration estimated using an evaporimeter (Evaposensor and Evapometer, Skye Instruments Limited, Powys, UK) in conjunction with gravimetric determination of water use. The drought stressed plants used for the diurnal experiment were given 50% of the irrigation supplied to the control plants for four weeks prior to sampling. During the two days of diurnal sampling the irrigation was switched off to prevent immediate response to available water. The drought stressed plants in the second experiment were given 25% (D25) and 0% (D0) of the irrigation supplied to the control plants. The Evaposensor was positioned amongst experimental plants at canopy height.

The substrate moisture content was measured three hours after irrigation, 20 h prior to sampling of the plants, by inserting electrodes of a probe (WET sensor, Delta-T devices, Cambridge, UK), deep into the root-zone from the surface. Stomatal conductance, *g*_S_ (mmol m^−2^ s^−1^) was measured three hours after sunrise, using a porometer (AP4, Delta-T Devices, Cambridge, UK) on two young, fully expanded leaves per plant. Whole plant transpirational water loss (ml h^−1^) was measured gravimetrically between irrigation times using a portable balance (AQT-5000 Camlab Limited, Cambridge, UK). Leaf water potential, *Ψ*_l_ (MPa) was measured on one fully expanded leaf per plant using a pressure chamber (Skye SKPM 1400, Skye instruments Ltd, UK).

### Sequence Analysis and Primer Design

The *F. vesca* genome sequence (available at the Genome Database for Rosaceae, (http://www.rosaceae.org/projects/strawberry_genome/v1.0/assembly) was queried to identify *PIP* aquaporins of *Fragaria*. Genomic sequences of *PIP* candidate genes were downloaded (File S1), start and stop codons, exons, introns and polyadenilation signals were assigned using the GENSCAN program available at the Massachusetts Institute of Technology (http://.genes.mit.edu/GENSCAN.html) and confirmed by alignment to *A. thaliana PIP* coding sequences. *F. vesca PIP* coding sequences were translated into protein sequences using the Translate Tool software (http://www.expasy.org/tools/) of the Swiss Institute of Bioinformatics. DNA and protein sequences were aligned with MAFFT software [45]. The transmembrane domains, intracellular and extracellular loops of the deduced protein sequences were identified using TMHMM software (http://www.cbs.dtu.dk/services/TMHMM/) of the Technical University of Denmark. *F. vesca PIP* genes were physically positioned on the pseudochromosomes using a blast search tool (http://www.rosaceae.org/node/1). Phylogenetic analysis included *A. thaliana* and *Zea mays* PIP sequences and was performed using PAUP* v4.0b10, using parsimony as the optimality criterion.

Primers were designed using Primer3 software [46]. Reverse primers for *PIP* isoforms were designed from the 3′UTR regions. Isoform specificity was tested by dissociation of amplification products in RT-qPCR and confirmed by PCR product sequencing.

### Harvesting Root and Leaf Material

For investigating diurnal expression, three biological replicates were taken per time point per treatment; each replicate comprised leaflets of equal size from three plants. The tissue was frozen in liquid nitrogen and stored at −80°C. Different sets of plants were sampled on two consecutive days. Leaves were sampled at two-hourly intervals between 04 h and 22 h. The sunrise was recorded at 06 h and the sunset at 20 h.

In the second experiment, leaf and root tissue of four plants per treatment was collected four hours after sunrise (06 h) on each day. Roots were sampled by taking the plant out of the pot, removing the perlite, briefly washing the roots and drying with tissue paper, wrapping in aluminium foil and freezing in liquid nitrogen. Manipulation of the roots during sampling was strictly under three minutes. The tissue was stored at −80°C.

### RNA Extractions and Reverse Transcription

RNA was extracted from root and leaf tissue of *F. vesca* in general accordance with the protocol of [47]. The quantity of RNA was determined with a spectrophotometer (NanoDrop 1000, Thermo Fisher Scientific). The integrity of RNA samples was tested by electrophoresis on a 1.4% agarose gel stained with ethidium-bromide. To reveal any residual genomic DNA contamination, PCR was performed with *Fragaria*-specific primers Fwd: caccggagtgtttcatgtcg and Rev: aacctccgaactgtctttgc as described in [48] using 100 ng of RNA sample as a template. RNA samples that amplified were considered contaminated and RNA was selectively re-precipitated as described in [47]. Reverse transcription was performed using Omniscript Reverse Transcription Kit (QIAGEN) starting with 100 ng of total RNA.

### Real-time qPCR

Five *F. vesca* candidate genes were evaluated as potential references. Gene expression stability validation was conducted in general accordance with [49]. Primers designed for five potential reference genes (eEF1α, GAPDH, Actin 7, serine/threonine protein phosphatase 2A regulatory subunit and 60 S ribosomal protein L21) were used in a validation run on the real-time PCR cycler, with eight cDNA samples representing different tissues and conditions used in the experiment (control leaf and root tissue, drought stressed leaf and root tissue, control and drought stressed leaf tissue sampled at midday and control and drought stressed leaf tissue sampled in the evening). The geNorm algorithm [49] was used to select the two most stable genes under the experimental conditions of this study: *FvGAPDH* and *FvEF1α*; these two genes could not be further ranked and had the gene expression stability measure *M* = 0.463. *FvGAPDH* was selected to be a reference gene.

Amplification efficiencies of aquaporin isoform-specific primers were compared individually to the efficiency of the *FvGAPDH* primers. The amplification efficiencies were high (90% ±5%) and comparable to the reference (difference <10%) and therefore relative quantities were determined using the ΔΔC_T_ method. qPCR reactions were performed in three to four biological replicates with three technical replicates for each sample. The final volume of each replicate was 20 µl comprising 4 µl of reverse transcription reaction, 1×SYBR® green master mix (Applied Biosystems) and 100–200 nM forward and reverse primers (Table 1). Reactions were performed on the 7500 Real Time PCR system (Applied Biosystems). The following cycling conditions were used: 50°C (2 min), 95°C (10 min), followed by 40 cycles of denaturation at 95°C (15 sec) and annealing and extension step at 60°C (1 min). Non-template controls were included in each run and all qPCR runs were followed by a dissociation stage and a single specific product was confirmed in every reaction. Average C_T_ values of the four genes were: 19.8 (*FvPIP1;1*), 22.18 (*FvPIP1;2*), 20.68 (*FvPIP2;1*) and 20.71 (*FvPIP2;2*).

**Table 1:** *F. vesca* PIP isoform-specific and reference gene primers.

Primer name	Forward primer sequence	Reverse primer sequence	Gene	Product length
FvPIP11	tgcagccatcatctacaacaag	gttgaaacgctcactcactgc	*FvPIP1;1*	165 bp
FvPIP12	gctgccatcatctacaacaagg	ccagcctagaagcaagtctaaatg	*FvPIP1;2*	183 bp
FvPIP21	caagacaaagcctgggatgacc	agcttgggtggaaaatcctg	*FvPIP2;1*	169 bp
FvPIP22	aatggatcttctgggttggac	tggaagcaacatctttcattgtg	*FvPIP2;2*	158 bp
FvGAPDH	tgggttacaccgaagatgatg	gcacgatcaagtcaatcacacg	*FvGAPDH*	168 bp

### Statistical Analysis

Statistical analyses were carried out using GenStat 9th Edition (VSN International Ltd.). Analysis of variance (ANOVA) tests were performed for each dataset with least significant difference (LSD) tests performed following ANOVA showing a significant effect (*P*<0.05). Diurnal expression analyses and transpiration per time of day were analysed by repeated measurements ANOVA. Correlation coefficients (*r*) were calculated to determine the significance of correlations between relative gene expression and the substrate moisture content for the corresponding plants. The strength of significant correlations were described as modest (*r = *0.40–0.69) strong (*r = *0.7–0.89) or very strong (*r = *0.90–1) [50].

## Results

### Identification and Characterisation of *F. vesca PIP* Sequences

**Figure 1:**
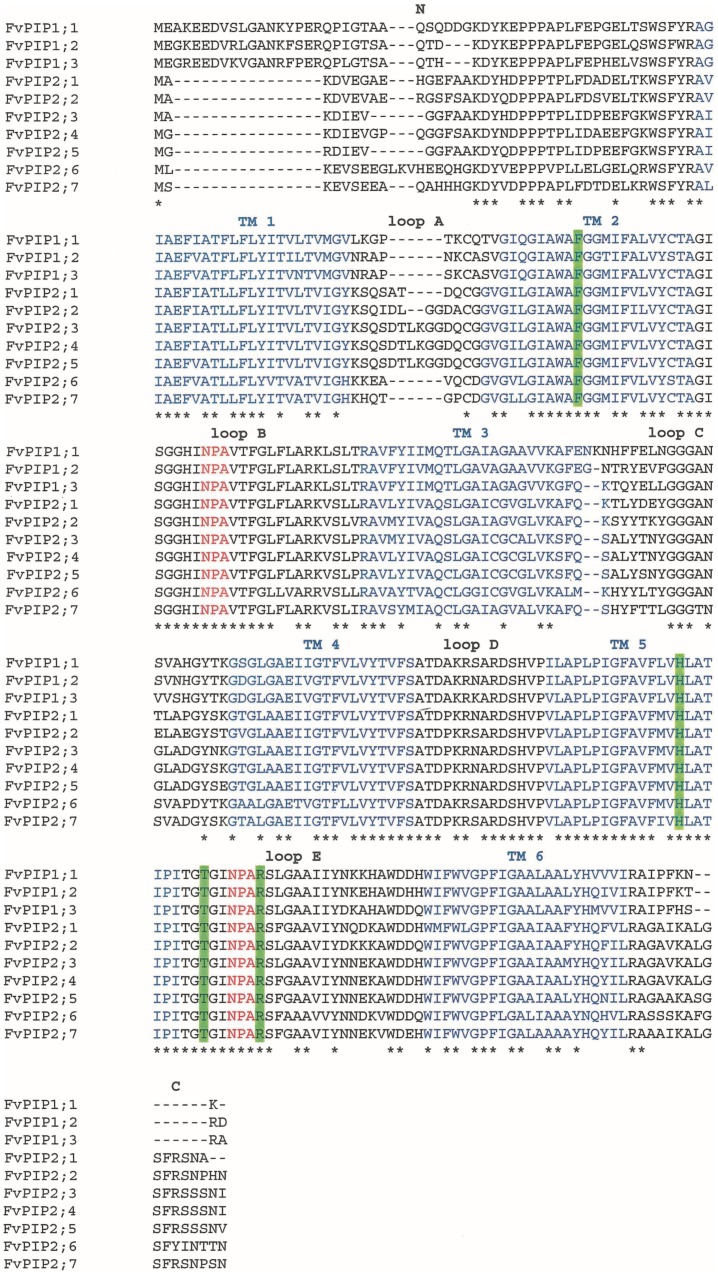
Deduced protein sequences of *F. vesca* PIP aquaporins. An alignment of *F. vesca* PIP deduced protein sequences. Blue – transmembrane domains (TM); red – NPA motif; green highlight – residues of the aromatic/arginine selectivity filter involved in determining water specificity. Asterisk denotes conserved sites. N stands for amino-terminal region and C stands for carboxy-terminal region of the protein.

*F. vesca PIP* aquaporins were identified and named according to existing aquaporin nomenclature [15]. Phylogenetic analysis of the deduced protein sequences identified three as PIP1 type and seven as PIP2 type aquaporins (Figure S2). Three members of the PIP2 subfamily, FvPIP2;3, FvPIP2;4 and FvPIP2;5 clustered together with 100% bootstrap support. The ten identified *PIP* sequences located to five *F. vesca* pseudochromosomes (Figure S3). Genes *FvPIP2;3 FvPIP2;4* and *FvPIP2;5* grouped closely together with only 8.5 kb between *FvPIP2;3* and *2;4* and 10.5 kb between *FvPIP2;4* and *2;5* on pseudochromosome six. The alignment of the *F. vesca* PIP sequences showed that *Fragaria* PIPs shared all the common structural features with other aquaporins (Figure 1). In addition, the four residues defining the constriction region of the aromatic/arginine selectivity filter, Phe (TM 2), His (TM 5), Arg (loop E) and Thr (loop E) were conserved in all sequences (Figure 1).

### Drought Stress Effect Prior to Diurnal Expression Analysis

**Figure 2:**
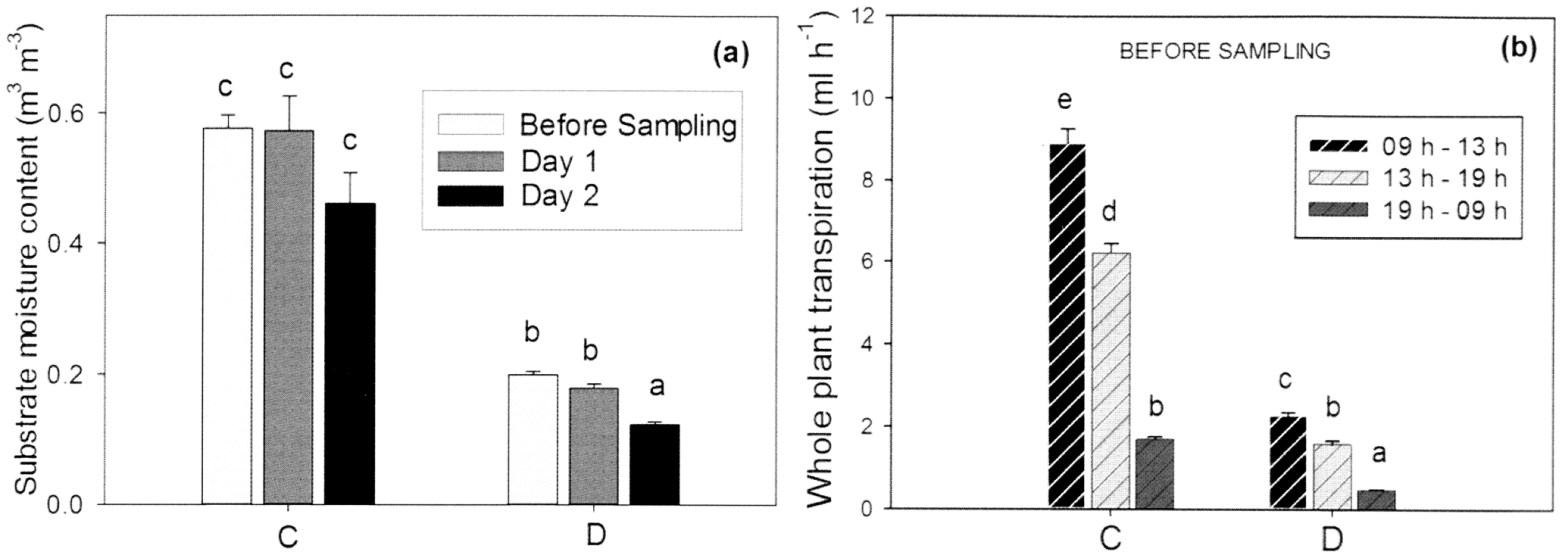
*F. vesca* water relations prior to diurnal expression analysis. Plots of *F. vesca* water relations showing (a) substrate moisture content and (b) plant transpiration rate per hour at different times of day. (C) control plants; (D) drought stressed plants. Data are means+SE, *n* = 18 plants. Different letters denote statistically significant differences determined by LSD following one way ANOVA for (a) and repeated measurements ANOVA for (b) (*P*<0.05).

Prior to diurnal sampling of leaves for expression analysis, *F. vesca* water relations were recorded. After four weeks of moderate water deficit the average volumetric substrate moisture content was 0.58 m^3^ m^−3^ in control plants and 0.22 m^3^ m^−3^ in the pots of the drought-stressed plants one day before sampling. During the diurnal sampling the irrigation was switched off and substrate moisture averaged 0.57 and 0.46 m^3^ m^−3^ in the control plants and 0.18 and 0.12 m^3^ m^−3^ in the drought stressed plants, on day one and day two, respectively. The difference in substrate moisture content of the drought stressed plants between day one and day two was significant (Figure 2a). Stomatal conductance after four weeks of treatments changed significantly from 356 mmol m^−2^ s^−1^ on average in the control to 95 mmol m^−2^ s^−1^ in the drought stressed plants and the whole plant transpiration rate was also significantly reduced from 4 ml h^−1^ on average in the control to 1.1 ml h^−1^ in the drought treatment. Both control and drought-stressed plants transpired significantly more water per hour between 09 h and 13 h compared to the rest of the afternoon, whilst the overnight transpiration was very low (Figure 2b).

### Diurnal Expression of *PIP* Aquaporins Under Normal Conditions and Water Deficit

**Figure 3:**
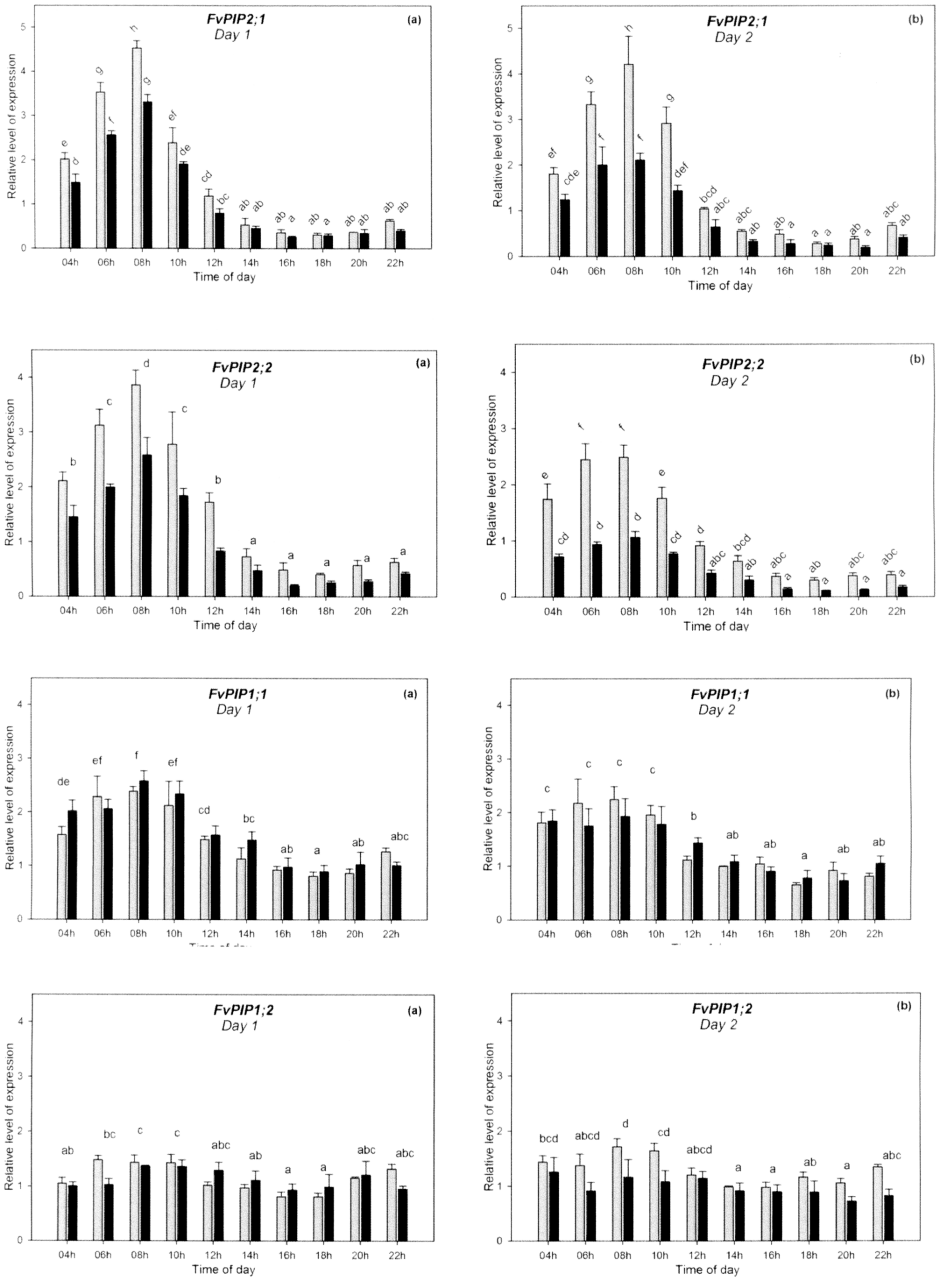
Diurnal expression of *F. vesca PIP* aquaporins. Diurnal expression pattern of *FvPIP* genes in leaves of *F. vesca* on two consecutive days. Grey columns – control plants, black columns – drought-stressed plants. Data are means+SE, *n* = 3 biological replicates. Different letters annotate statistically significant differences determined by LSD following repeated measurements ANOVA (*P*<0.05). The difference between treatments was significant for (a), (b), (c), and (d).

Diurnal expression analysis revealed that three of the genes showed a distinct diurnal pattern, consistent between the two days (Figure 3). The transcription of *FvPIP2;1* in leaves of control plants peaked 2 h after sunrise (08 h) and the transcript abundance decreased more than fourteen-fold between the peak time and the lowest point (18 h). The plants subjected to four weeks of water stress showed a relatively similar pattern of diurnal expression: a peak two hours after sunrise and a sustained down-regulation throughout the afternoon hours on both days, with over eleven-fold difference between the peak time and the lowest point (Figure 3a,b). However the transcription was significantly reduced in the drought stressed plants during the morning, whilst between 14 h and 22 h the expression in both control and drought stressed plants was low and the differences were not significant. Gene *FvPIP2;2*, also showed a marked diurnal expression very similar to *FvPIP2;1* (Figure 3c,d). The difference in transcript abundance between the highest and the lowest expression level was more than eight-fold in both the control and the drought-stressed plants. The gene *FvPIP1;1* was more abundantly expressed in the morning with the peak of expression at around 08 h followed by a significant down-regulation with around three-fold reduction in transcript abundance (Figure 3e,f). No significant effect of the imposed drought-stress on the expression of *FvPIP1;1* was observed in the leaf. The gene *FvPIP1;2* did not show a marked diurnal rhythm although the *FvPIP1;2* transcript was more abundant at 08 h and 10 h than in the afternoon hours (14 h, 16 h and 18 h) on both days (Figure 3g,h). This gene did not respond significantly to the drought-stress treatment on either of the days.

The drought stress showed an intensity dependent effect on the diurnal expression: namely, the plants were not irrigated during diurnal sampling which caused substrate moisture content to decline slightly on the second day and the difference was significant in the pots of drought stressed plants (Figure 2). This decrease in substrate moisture was accompanied by a reduction in the amplitude of expression of both *FvPIP2;*1 and *FvPIP2;2* (Figure 3b,d). The light conditions were similar on the two mornings (Figure S1).

### *F. vesca* Water Relations under Different Levels of Drought-stress

**Figure 4:**
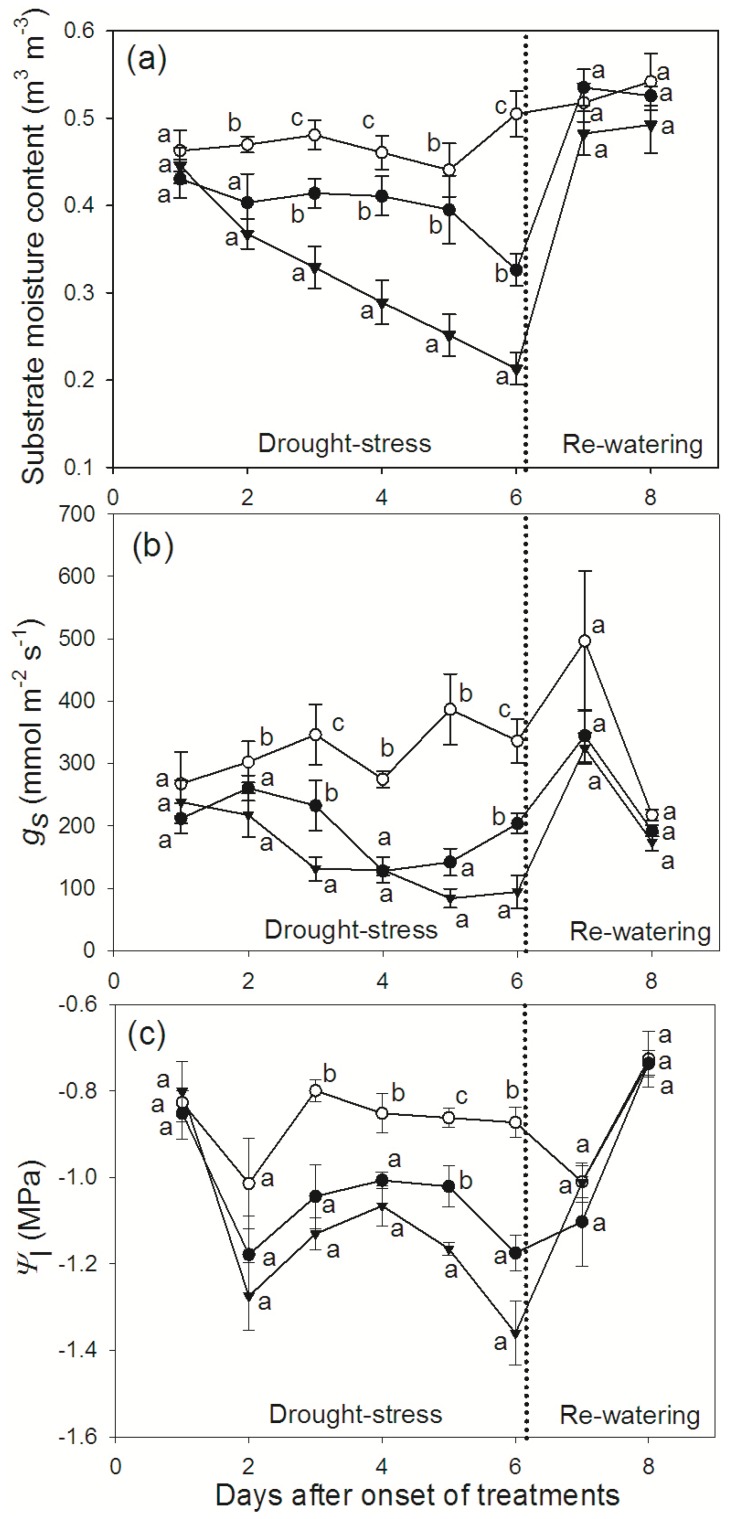
*F. vesca* water relations under different levels of water-stress. Plots showing (a) substrate moisture content, (b) stomatal conductance and (c) leaf water potential under different levels of water-stress. Open circles – control plants; filled circles – plants receiving 25% of the control irrigation; filled triangles – plants with no irrigation. Data are means ± SE, *n* = 4 plants. Different letters denote statistically significant differences on a given day, determined by LSD following one way ANOVA (*P*<0.05).

In order to investigate the effects of different intensities of drought stress on *PIP* expression, *F. vesca* plants were subjected to two levels of water deficit (D25 and D0) alongside a control group, for the duration of six days. The average substrate moisture content was reduced by 34% and 58% compared to the control, in D25 and D0 treatments respectively, on day six. The recorded stomatal conductance on the same day was reduced by 40% and 72% compared to the control, whilst leaf water potentials decreased by 33% and 56%, in the D25 and D0 plants respectively. Upon re-watering (days seven and eight), both stomatal conductance and leaf water potentials recovered to control levels (Figure 4).

### *PIP* Expression in Roots and Leaves under Different Levels of Water Stress

**Figure 5:**
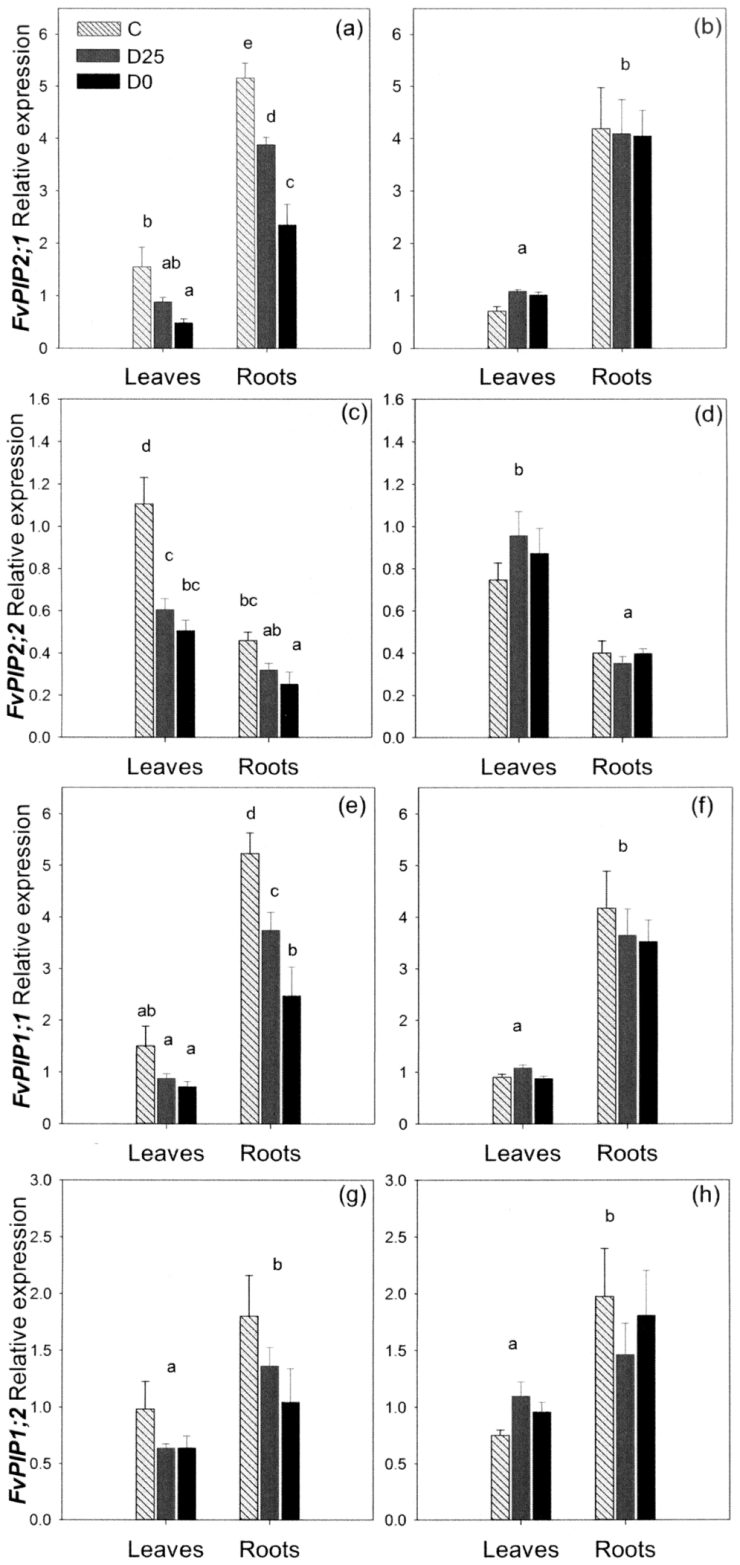
Relative *PIP* expression in leaves and roots of *F. vesca* under different levels of drought stress. Expression of four *F. vesca PIP* aquaporin genes after six days of drought stress (a, c, e, g) and upon re-watering (b, d, f, h). C – control plants; D25– plants receiving 25% of the control irrigation; D0 − plants with no irrigation. Data are means+SE, *n* = 4 plants. Different letters denote statistically significant differences within each time point determined by LSD following one way ANOVA (*P*<0.05).

C_T_ values showed that the expression of *FvPIP1;2* was the lowest; 3–4 times lower than the expression levels of the other three genes. The most abundantly expressed gene in the roots was *FvPIP1;1* followed by *FvPIP2;1*, whilst in the leaves it was *FvPIP2;2* that was most abundant. The gene *FvPIP2;1* showed significantly higher transcript levels in roots compared to leaves (Figure 5a,b). By day six of water stress, D0 plants reduced the abundance of the *FvPIP2;1* transcript significantly in the leaves, whilst the down-regulation in the D25 treatment remained non-significant. In the roots, however, both treatments were significantly different to the control and to each other. Aquaporin transcription was up-regulated back to control levels in both groups of drought-stressed plants upon re-watering.

The *FvPIP2;2* gene showed significantly higher transcript abundance in the leaves compared to the roots (Figure 5c,d). After six days of drought stress the level of *FvPIP2;2* expression was significantly reduced in the leaves of plants in both the D0 and D25 treatments, whilst the difference in roots was significant for the non-watered plants only. Expression levels were restored in both leaves and roots upon re-watering.

The *FvPIP1;1* gene showed a higher expression in the root than in the leaves and expression in leaves was not significantly altered by drought-treatment (Figure 5e,f). However, the plants responded to six days of water-deficit by significantly reducing transcript abundance in roots. Transcript abundance was intermediate in the D25 treatment and significantly different to both the non-watered plants and to the control. The transcription level in the roots returned to normal after re-watering.

The gene *FvPIP1;2* was expressed in both root and leaf tissues (Figure 5g,h). On average, the expression level in the roots was higher than in the leaves, but the differences were not as clear as for the other three genes: Namely, whilst in most of the plants the transcript abundance was higher in the root, in some plants the transcript was more abundant in the leaves. In addition, even though some changes in expression were apparent, no statistically significant trends in response to water-stress could be identified.

### Correlation between Substrate Moisture and *PIP* Expression

**Figure 6:**
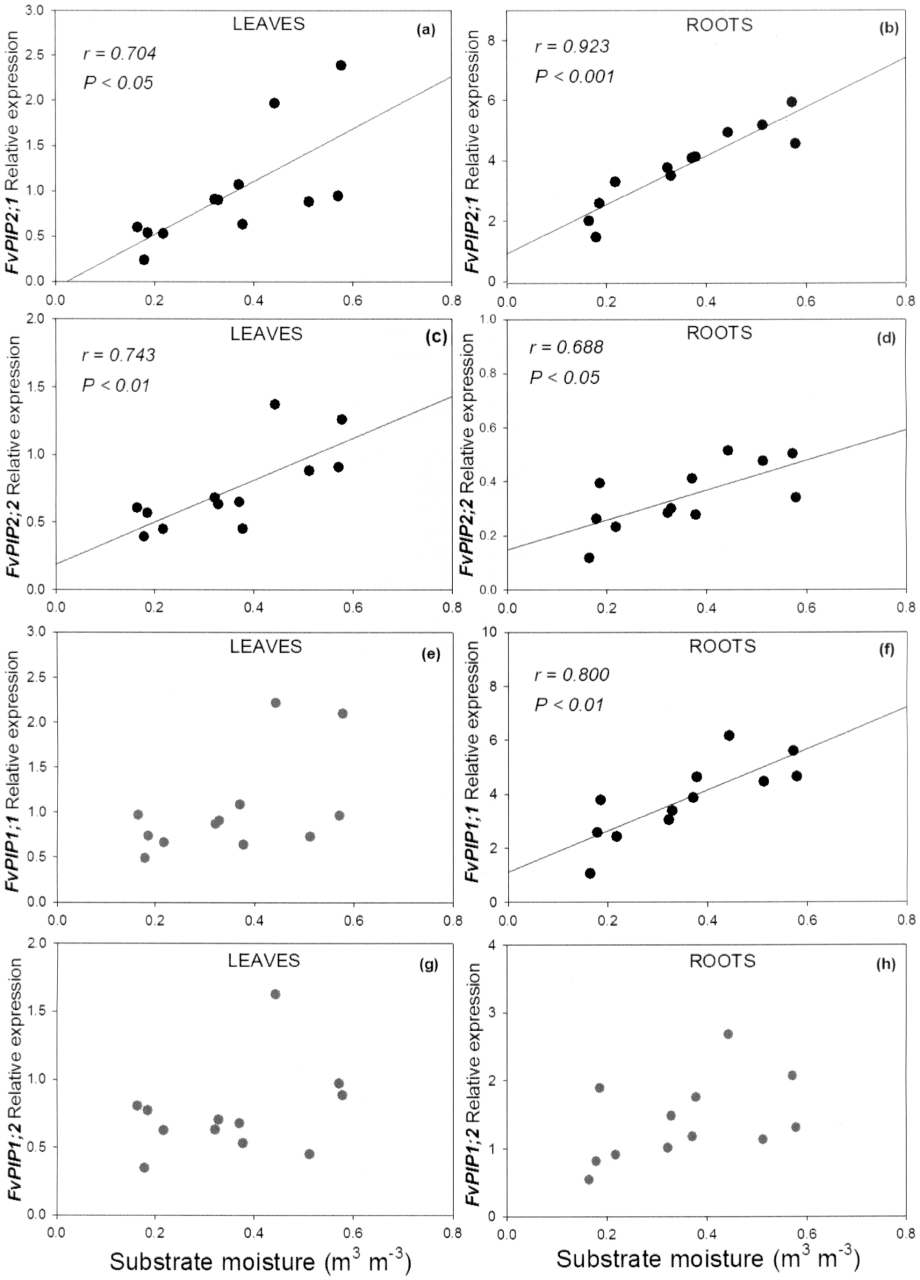
Correlation between substrate moisture content and relative expression of *FvPIP* genes. *FvPIP2;1* (a, b), *FvPIP2;2* (c, d), *FvPIP1;1* (e,f), *FvPIP1;2* (g, h) in leaves (a, c, e, g) and roots (b, d, f, h). Regression lines, correlation coefficients and probabilities are given for statistically significant relationships.

The expression of *FvPIP2;1* in the leaf tissue was strongly correlated (*r = *0.704), and the expression in the root tissue was very strongly correlated (*r = *0.923) with the substrate moisture content (Figure 6a,b). The transcription of *FvPIP2;2* was correlated strongly in leaves (*r = *0.743) and modestly in roots (*r = *0.688) (Figure 6c,d). *FvPIP1;1* showed the relative quantity of transcript in the root tissue to be strongly correlated to the substrate moisture content (*r = *0.800), whilst the expression in leaves showed no significant correlation with substrate moisture content (Fig. 6e,f). The transcript abundance of *FvPIP1;2* in both leaves and roots was also not correlated to the substrate moisture content (Fig. 6g,h).

## Discussion

### Fragaria *PIP* Genes

Only five *PIP* isoforms have been characterised from the Rosaceae family so far [35] [51] [52] [53]. In the *F. vesca* genome ten *PIP* aquaporins were identified, allowing a more complete investigation of the *PIP* subfamily from a species within the Rosaceae. Sequences showed a structure typical of plant PIP aquaporins; all had the residues of the aromatic/arginine selectivity filter conserved, pointing to water selectivity of these isoforms [19]. The ten genes were spread over five chromosomes, with the exception of *FvPIP2;3, FvPIP2;4* and *FvPIP2;*5 which grouped closely together. Due to their close physical proximity, high degree of sequence homology and phylogenetic relatedness, it is likely that these three *F. vesca PIPs* have arisen by gene duplication as a result of unequal crossing-over.

The *F. vesca FvPIP1;1* deduced protein sequence showed a very high (99%) homology to the previously reported *F.*×*ananassa FaPIP1;1*, which was found to be involved in fruit ripening [52], implying that the two genes may be orthologs. Only two amino acids were found to be different – one in the N-terminal region and one in the transmembrane domain TM1. Interestingly, the expression patterns of the two genes seem to be very different. Mut *et al.* [52] found *FaPIP1;1* to be expressed in ripe fruit and ovaries but no transcript was detected in leaves or roots. In contrast, the present study showed that the putative ortholog from *F. vesca*, *FvPIP1;1*, is expressed in leaves and highly expressed in roots. In the light of these findings it would be interesting to study the promoter regions of these two genes. *F.*×*ananassa* is an allo-octoploid species and may contain multiple copies of a single isoform, some of which may be inactive and others could potentially have evolved to perform different roles. It also cannot be excluded that the minor differences in the protein sequence could elicit some functional changes.

### *FvPIP* Aquaporins under Drought Stress

Three of the four *PIP* genes investigated in this study were found to be expressed in a diurnal pattern and the same genes were also significantly down-regulated by drought stress. *FvPIP2;1* and *FvPIP2;2* responded in both leaves and roots, whilst *FvPIP1;1* responded only in the root system (drought stress had no significant effect on its expression in the leaves) implying differential regulation in the two tissues. The fourth gene investigated, *FvPIP1;2*, had no clear diurnal pattern of expression and was unaffected by drought stress; in addition this gene also had the lowest expression of the four *PIPs* investigated.

#### *FvPIP* diurnal expression in leaves and the effect of drought stress

Clear daily fluctuations of aquaporin expression in the leaf were observed for *FvPIP1;1*, *FvPIP2;1* and *FvPIP2;2* and showed a similar general profile of expression for the three isoforms, with a peak two hours after sunrise, a reduction of transcription there onwards and recovery towards the end of the night period.

There have been no reports thus far on how drought stress affects the diurnal fluctuation of aquaporin expression. The moderate drought stress imposed in this study changed the abundance of *FvPIP2;1* and *FvPIP2;2* transcripts whilst the patterns of diurnal expression remained similar to the control – the peak of expression did not disappear, there were no additional peaks and the expression did not shift towards earlier or later in the day. Significant differences in expression between stressed and control plants were generally observed in the morning hours, when the aquaporin expression was high. Additionally, there was an intensity dependent effect on the diurnal expression between the two days of diurnal sampling in the drought stressed group.

The observed peak of aquaporin expression occurred just before the highest transpiration levels of *F. vesca.* Assuming that there is a lag between aquaporin transcription and enhanced aquaporin activity in the membranes, the timing of the diurnal transcription in *F. vesca* could be a response related to daily peaks in transpiration. It is important to note that under drought stress the diurnal pattern persisted, although attenuated, which was consistent with the transpiration still being significantly higher during late morning and midday (Figure 2b). Diurnal variations in root hydraulic conductance, found to be accompanied by variation in abundance of *PIP* transcripts are considered to have an effect of reducing xylem tensions at high transpiration demand [30]. In addition it has been suggested that enhanced activity of leaf aquaporins during the day may favor transport into the inner leaf tissues during maximal transpiration, which would prevent very low leaf water potentials and reduce xylem tensions [30]. New insights in rice imply that rapid up-regulation during *PIP* diurnal expression in this species may be caused by a signal from the shoots arising from increased transpirational demand after light initiation [31].

#### Correlation between *FvPIP* expression and substrate moisture content

In a study on the whole family of *Arabidopsis* aquaporins, Alexandersson *et al*. [37] investigated the effect of drought-stress and suggested that it may be necessary to stop aquaporin synthesis at levels of drought below 30% of soil water content in order to minimise water flow through cell membranes and prevent further water loss. Our results show that the response of some aquaporins to drought stress may be more gradual and fine-tuned. Under water-stress, the transcript levels of *FvPIP2;1* and *FvPIP1;1* in the root were reduced in a quantitative manner reflecting the severity of the stress. In fact, the transcription of *FvPIP2;1* and *FvPIP1;1*, both highly expressed in the root, was strongly correlated to the substrate moisture content as it declined from nearly 60% to under 20%. The *FvPIP2;2* transcript was moderately correlated to the substrate moisture content when analysed in the root and strongly in the leaf where it was more abundant.

When investigated in relation to distant parts of the plant such as leaves, the soil moisture can only be viewed as a good measure of the imposed stress and it is hard to imagine a direct impact of this parameter to gene expression in distant organs. However, the impact on the root cells of the surrounding substrate drying is far more immediate. Roots have a remarkable capacity to sense physico-chemical parameters of the soil and adjust their transport properties accordingly and they play a central role in maintaining the water status of the whole plant in a changing environment [11]. The question therefore is how the substrate moisture content is monitored by the plant and which processes might be involved in substrate moisture perception and *FvPIP* response in the root.

In the present study, the osmotic potential of the feeding/irrigation solution applied to a homogenous and inert perlite medium was the same for all plants, as was the effect of gravity and external (atmospheric) pressure. As soil water potential depends on four components – matric and osmotic potentials, gravitational force and external pressure [2], a sensory mechanism affecting *FvPIP* expression in *F. vesca* roots would have to be sensitive primarily to the changes in the matric potential (*Ψ*_m_) and the differences in surface tension it may create during substrate drying.

Aquaporins in the membrane are likely to be under control of osmo- and pressure-sensing molecules and downstream signalling cascades [30]. It has been proposed that the activity of aquaporin proteins, known to be affected by Ca^2+^ dependent phosphorylation, could be controlled by stretch-activated Ca^2+^ channels functioning as osmosensors and responding to water potential changes in the apoplast [57]. In addition, aquaporin tetramers themselves have been postulated to function as osmo- and pressure-sensing molecules [58] [59]. These hypotheses however, aim to explain gating of aquaporins, whilst the present study suggests that some sensory mechanism must be affecting aquaporin transcript abundance as well.

Aquaporin isoforms exhibit a diverse range of responses to stress involving both ABA-dependent and ABA-independent signalling pathways [36] [54]. Hachez *et al.* [10] proposed a division of aquaporin isoforms into constitutive and stress-responsive, the former of which would be down-regulated during drought and salt stress as plants try to avoid excessive water loss, whilst the latter would be up-regulated (or show stable expression) in order to perform specific roles in the plant under stress. A comprehensive study using a multi-level approach in maize showed that ABA affects gene expression and protein abundance of most *PIP* isoforms in the root by increasing expression rather than through down-regulation [60] and similar results were found in leaves and roots of Arabidopsis and rice [36] [55]. On the other hand, Alexandersson *et al.,* [39] showed that in Arabidopsis, many *PIP* and *TIP* genes that are down-regulated upon drought stress are strongly co-expressed and that most of the *PIP* transcriptional variation during drought stress could be explained by one variable linked to leaf water content. In our study, the down-regulation of aquaporin expression in the root tissue was strongly correlated to the declining moisture content of the surrounding substrate, and therefore it would be tempting to speculate that the down-regulation of *PIP* aquaporins, which has been frequently observed in response to drought stress [23] [34] [36] [37] [39] but at odds to the trend shown for the effect of ABA [36] [54] [55] [56], might perhaps be linked to a ubiquitous hydraulic or osmotic signal generated as the surrounding water potential declines. However, further studies need to be conducted in order to distinguish between potentially different pathways of regulating aquaporins under drought stress and also to determine the mechanisms underlining the correlation between moisture content and the expression of these highly responsive genes.

## Supporting Information

Figure S1 The average and maximum photosynthetically active radiation (PAR) recorded in the controlled environment compartment (a) during the diurnal experiment (b) during the experiment with two levels of drought stress.
(JPG)

Figure S2 **Phylogenetic analysis of** ***F. vesca*** **PIP aquaporins with** ***A. thaliana*** **and** ***Z. mays*** **PIP sequences.** The numbers represent bootstrap values. Branches encircled in red and blue represent the PIP1 and PIP2 clades respectively.
(JPG)

Figure S3 **Physical positions of** ***FvPIP*** **aquaporin genes on the seven** ***Fragaria*** **pseudochromosomes (FC1–FC7).** The physical distance is denoted by numbers where 1 = 100 kb.
(JPG)

File S1 ***F. vesca PIP*** **genomic sequences and CDS.** Gene annotation follows that of the Strawberry Genome Version 1.0 release.
(TXT)
