# Supplementary figures and images for: Expression of Fragaria vesca PIP Aquaporins in Response to Drought Stress: PIP Down-Regulation Correlates with the Decline in Substrate Moisture Content
Source: PLoS One. 2013 Sep 23;8(9):e74945. doi: 10.1371/journal.pone.0074945 (PMC3781111; doi:10.1371/journal.pone.0074945)

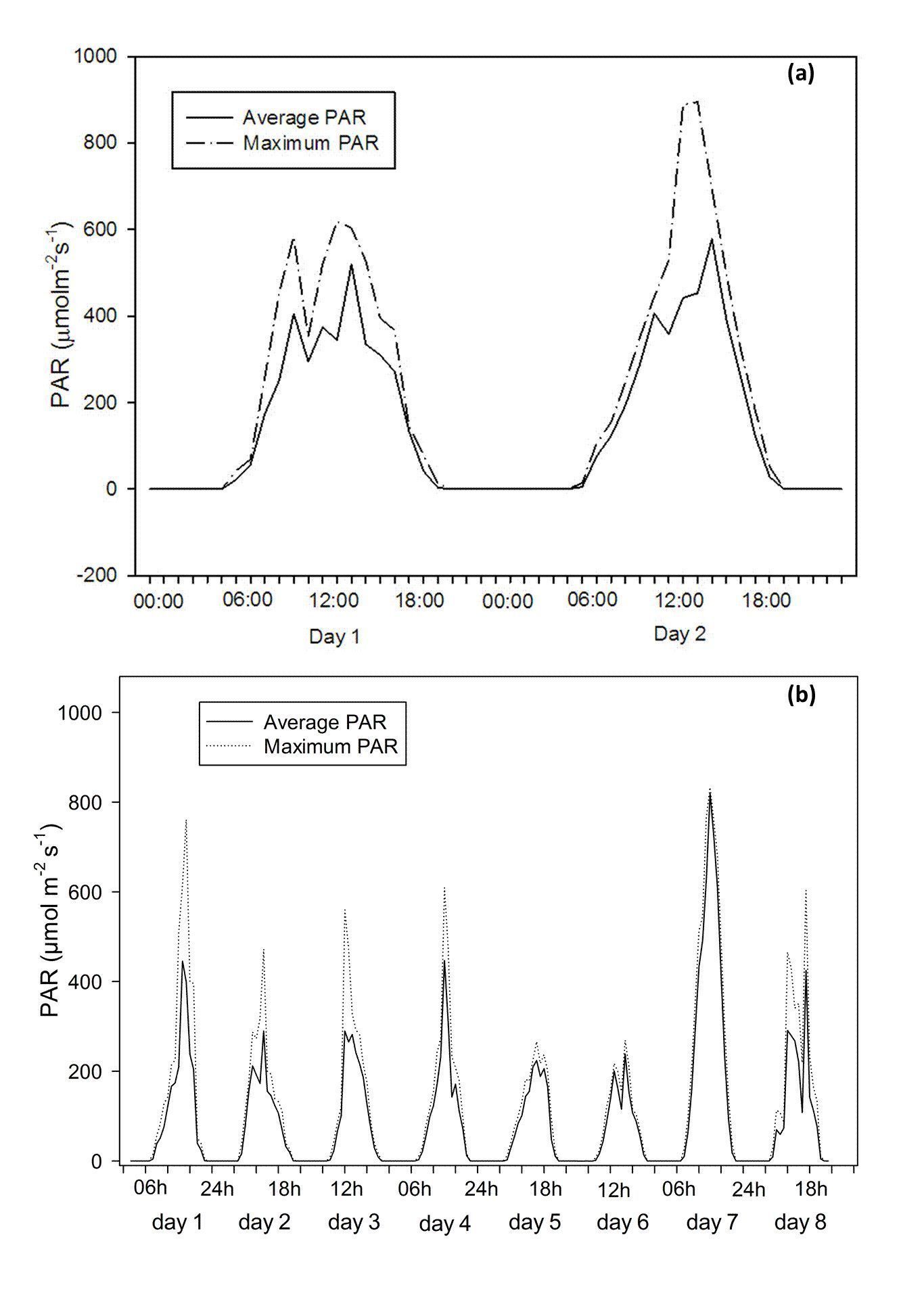

Supplement: Figure S1 — The average and maximum photosynthetically active radiation (PAR) recorded in the controlled environment compartment (a) during the diurnal experiment (b) during the experiment with two levels of drought stress. (JPG) [file pone.0074945.s001.jpg]

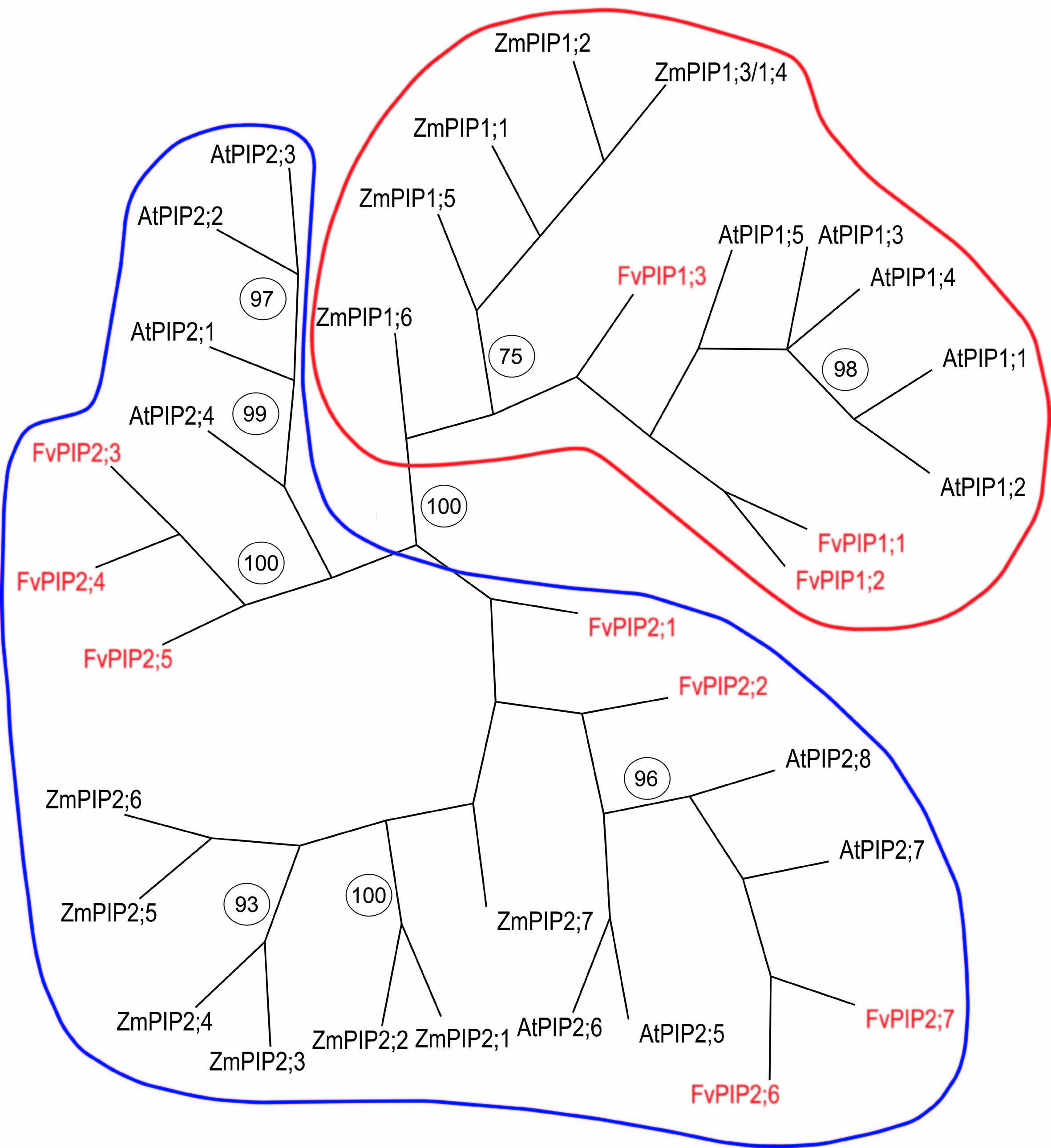

Supplement: Figure S2 — Phylogenetic analysis of F. vesca PIP aquaporins with A. thaliana and Z. mays PIP sequences. The numbers represent bootstrap values. Branches encircled in red and blue represent the PIP1 and PIP2 clades respectively. (JPG) [file pone.0074945.s002.jpg]

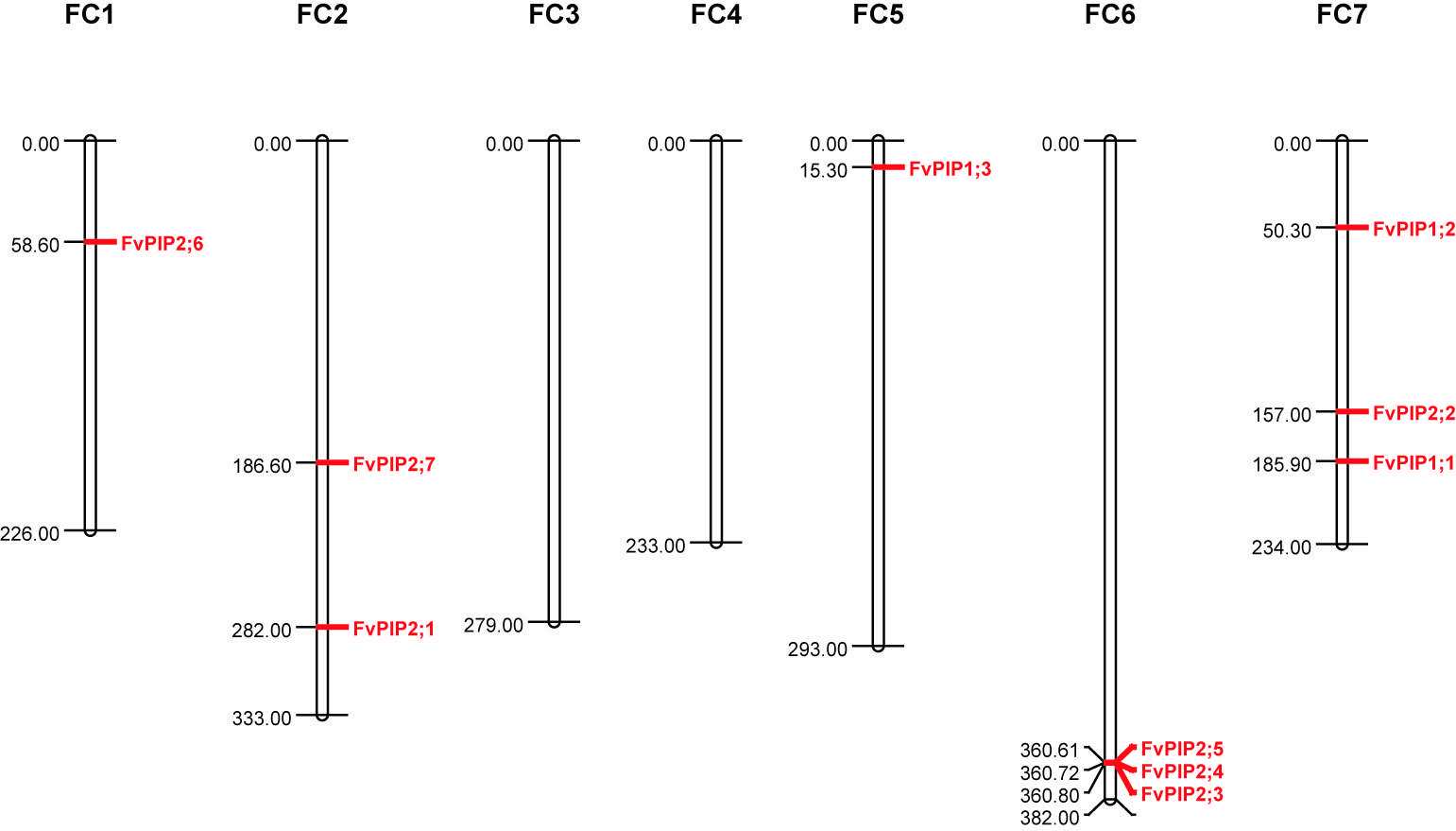

Supplement: Figure S3 — Physical positions of FvPIP aquaporin genes on the seven Fragaria pseudochromosomes (FC1–FC7). The physical distance is denoted by numbers where 1 = 100 kb. (JPG) [file pone.0074945.s003.jpg]
